# Prophylactic Aspirin and Public

**DOI:** 10.3934/publichealth.2013.1.1

**Published:** 2013-10-31

**Authors:** Gareth Morgan

**Affiliations:** NHS Wales Policy Lead for Older People, NHS Wales, 13 Goring Road, Llanelli, Carmarthenshire, Wales, SA15 3HF, UK. E-mail: Gareth.Morgan5@wales.nhs.uk.

**Keywords:** aspirin, public health, vascular disease, cancer

## Abstract

The clinical use of aspirin, first synthesized over 100 years ago, entered a new phase in 1974 with the reporting of the first randomised trial showing a reduction in vascular disease deaths from low-doses. More recent evidence suggests that the medicine is effective against cancer, which makes aspirin of very considerable potential importance to public health improvement and potentially also to clinical practice. It appears that prophylactic aspirin is being increasingly used throughout the community. There is need therefore for the risks and benefits of low-dose aspirin, and its' role within healthcare and within public health, to be widely discussed not least as media reports are bringing this issue into the public domain. It also follows that policy decisions need to be taken as to whether or not its use should be actively promoted. In particular, it is important that Doctors and healthcare practitioners are well informed of the risks and benefits so that they can impart this knowledge during consultations. Furthermore, it is important that low-dose aspirin is not perceived as a substitute for a healthy lifestyle, but that it is recommended and uptake monitored alongside other protective behaviours to improve on health gain, such as smoking cessation, moderate alcohol intake, exercise and diet.

## 1. Introduction

Aspirin is an inexpensive, easily available, well-known and widely used medicine. Many households will have this medicine or products containing it, such as is the versatility of aspirin in treating pain, inflammation and fever. The medicine also has other benefits, including the reduced risk of vascular disease and emerging evidence supports a role in cancer prevention. The benefits, however, also need to be balanced against the risks of bleeding caused by aspirin and set in the context of other measures to improve health and reduce disease risk.

In order to tackle some of these questions, a Citizens' Jury was conducted in 2006, under the title: “My Health—whose responsibility?” [1]. A verdict of the sixteen jurors, who had been chosen by quota sampling to be representative of the general public, was that while the treatment of disease is delegated to healthcare practitioners, the prevention of disease is the responsibility of each individual person. In relation to the preservation of health, the responsibility of healthcare practitioners is therefore to make information on healthy behaviours readily available and accessible to the public. Further, the jurors also added that “departments of public health should take a key role in achieving this”. The role of aspirin is particularly relevant to this and perhaps it may be considered a fifth healthy behaviour.

The presentation of evidence on healthy behaviours to the Jury included information on low -dose aspirin as a medicine that helps to preserve health. At the time of that jury, the evidence of benefit from aspirin was limited to vascular disease and evidence on a reduction of cancer was only suggestive. Yet the jurors stated that information on the risks and possible benefit of aspirin on cancer risk should be made available to the public “…even before there is agreement amongst doctors”. Debate still remains among the medical profession about the role of aspirin in healthy ageing.

Evidence on the benefits of aspirin in the reduction of cancer risk has very greatly increased in recent years and so, in partial response to the verdict of the convened Jury, this paper summarizes the present evidence on the benefits and risks of low-dose aspirin. Further to this, discussion is then offered on the balance between benefits and risks of the medicine, with an interpretation of this for public health policy. The ongoing debates on this topic are also acknowledged and discussed.

## 2. Aspirin and Vascular Disease

Low-dose aspirin prophylaxis, 75-150 mg per day, is now accepted for all those who have clinical evidence of vascular disease, together with those individuals who have a raised vascular risk score [2]. However, there is evidence of considerable under-use of aspirin prophylaxis in such patients [3], due to both poor compliance and undesirable effects of aspirin. Boosting the use of aspirin prophylaxis in those who have been recommended the medicine by a healthcare professional could yield population benefit by reducing the number heart attacks and ischemic strokes [4]. As will be discussed later, aspirin reduces the risk of ischemic strokes caused by clots but increases the risk of those strokes caused by bleeding.

Aspirin is not generally recommended for healthy individuals on the grounds that the number of vascular events likely to be prevented is similar to the number of gastrointestinal bleeds likely to be precipitated. Such evaluations are based on the assumption that a bleed can be equated in severity with a heart attack or an ischemic stroke. With regard to severity and consequence this can be challenged and a comment in one of the major overviews of aspirin trials is apposite to this: “…the alternative to primary prevention is deferral until some evidence of occlusive disease is noted… but the first manifestations of disease might be a disabling or fatal event” [4]. Yet, do the general public appreciate this and the relative risks and benefits of taking aspirin.

Furthermore, this situation brings into question what is meant by the term “healthy”. Many people living in the community will have undiagnosed vascular disease so aspirin use could therefore have a substantial beneficial impact. It should also be recognized that the use of statins is also a relevant consideration in this situation. As for aspirin, the evidence on cancer is a further element to this equation on the balance of benefits and risks. In the last 5 years, the evidence has strengthened considerably on aspirin and cancer.

## 3. Cancer

Evidence from many sources which suggested a reduction in cancer [5] has now been supplemented by the results from patient data from long-term follow-up of twenty-five thousand individuals who had participated in early randomised trials of vascular protection by aspirin [6],[7]. Analyses on an “intention to treat” basis of data from eight trials showed that starting about five years after daily low-dose aspirin had commenced, there was a reduction of one third in all cancer (Hazard ratio (HR) 0.66; 95% confidence limits (CI) 0.50, 0.87) and a 10% reduction in all-cause deaths. The reductions were maintained 20 years later in three trials which had adequate data and benefit increased with duration of aspirin taking and also with increasing age. This finding has important and far-reaching public health implications, not least due to the challenging financial pressures on healthcare systems and the increasing demands related in part to the ageing population. Further evidence of benefit comes from an ad hoc trial of aspirin given to patients with Lynch syndrome, the major form of hereditary bowel cancer [8]. Patients who had completed two years on aspirin had a relative reduction (RR) in incident bowel cancer (RR 0.37; 0.18, 0.78) and other cancers. As well as these studies, which do not give final answers, from the general population and genetically predisposed individuals, there is also evidence aspirin might be used as an adjunct treatment of cancer [9]. This suggests aspirin might be exerting effects on a wide range of cancer pathways and at a variety of stages in the carcinogenic pathway. There remain questions to be answered but the potential public gain is considerable from appropriate aspirin usage.

So how can this potential be harnessed for public health gain? As a practical approach, the potential of aspirin exists to enhance bowel cancer screening [10]. Indeed, experience suggests that the uptake of bowel cancer screening is not 100% and for those individuals not taking it up or in the countries where the programme does not exist, perhaps aspirin has an important health gain role to play.

## 4. The Risks of Aspirin Prophylaxis

In six primary randomised trials [11], the risk of death from gastrointestinal bleeding in the subjects receiving aspirin (4 per 100,000 subjects per year) was almost identical to that in subjects who had been allocated to placebo (5 per 100,000 per year). The report of a previously cited meta-analysis [2], based on 660,000 follow up years, states: “…there were actually fewer fatal bleeds in participants allocated to aspirin than in the controls (nine vs twenty)”.

There is also evidence from trials that the incidence of bleeding goes down over time, which may have important implications for identifying early individuals with particular risk factors. An overview of studies found that the risk of a gastrointestinal bleed was highest during the first month of aspirin use [6]. While it can be argued that this reduction in bleeding over time could be due to stopping aspirin, or taking a gastric acid suppressing drug along with the aspirin, it can again be said that these measures represent good clinical practice and could be matched in the community if doctors and individuals are also adequately informed. Again, the question of accurate information is relevant.

More seriously, aspirin increases the risk of a haemorrhagic stroke [12]. Two or three individuals in every 10,000 taking aspirin may experience a cerebral bleed. The major risk factor for stroke is hypertension, and there is evidence, albeit limited, that cerebral bleeds attributable to aspirin may occur mainly, or perhaps only in those with inadequately treated hypertension [13]. Many of the guidelines on aspirin prophylaxis therefore recommend that blood pressure is checked, and adequately treated if raised, before aspirin is commenced. Part of the challenge here is that hypertension in the community might be either undiagnosed or poorly managed. So there are challenges to be overcome given the devastating effects of a haemorrhagic stroke.

## 5. Who should Take Prophylactic Aspirin?

One of the challenges in converting the public health potential of aspirin into a reality is to boost the under-use of the medicine in those individuals with a history of vascular events. Whilst acknowledging the wider use of medication compliance, this discreet situation could be improved by strategies such as information campaigns and perhaps supporting those individuals with a history of a vascular event with self-management strategies. On the basis of vascular risk alone, recommendations have been made [14] and challenged [15] that regular aspirin taking be considered by subjects over the age of about 50 years. Others suggest that the benefits of aspirin on cancer risk ‘will tip the balance in favour of treatment’ of healthy older subjects and they recommend aspirin from the age of about 45 years [6],[7]. On the other hand, the evidence available at present is inadequate to fix an upper age limit for vascular or cancer prophylaxis and it seems unfortunate that estimates of the likely risks and benefits in people of advanced years are based on results of trials which included few elderly subjects. This situation remains a strongly debated issue.

Aspirin has been shown to reduce the number and the growth of bowel adenomas [16],[17] and prophylaxis would therefore seem to be especially appropriate alongside bowel screening activities. While the taking of low-dose aspirin may increase false positive results from the faecal occult blood (FOB) test, the sensitivity of immunochemical FOB tests is ‘markedly increased’ in subjects taking low-dose aspirin [18]. Further, while the distal bowel can be relatively easily examined by sigmoidoscopy, aspirin is associated with a reduction in proximal bowel cancer. So aspirin could complement rather than duplicate bowel cancer screening programmes.

## 6. Social and Public Health Issues

Low-dose aspirin is preventive, both against vascular disease and against cancer. It is not treatment and should perhaps be considered alongside the healthy behaviours. If low-dose aspirin is promoted within the context of the preservation of health, the risks and benefits should be clearly explained so that individuals are enabled to make informed decisions about the protection of their own health. It should be noted, however, that there may be unintended consequences of wider aspirin use, namely individuals do not make other lifestyle changes. There may be an opportunity to therefore re-brand aspirin for primary prevention is ways similar to the taking of vitamins.

It needs to be noted, however, that the prevalence of aspirin taking throughout the community is high. In a recent survey in south Wales 30% [3] of persons aged over 50 years reported regular aspirin taking and half were taking an unnecessarily high dose. In the USA, 36% of the adult population have been estimated to take regular aspirin [18]. These proportions are likely to increase as knowledge of the effect of aspirin on cancer spreads, and it is important therefore that informed debate is stimulated on the risks and benefits of prophylactic aspirin and on how it is to be handled within healthcare.

Regarding the stopping of aspirin prophylaxis, it should be born in mind that aspirin withdrawal is associated with a rebound in vascular disease risk. More dramatic results come from a small randomised study in which subjects who bled while on low-dose aspirin were all given a gastric acid suppressant and then half were randomised onto aspirin again or to a placebo. All-cause mortality was 1.3% in those put back on aspirin and 10.3% in those on placebo [20]. It could be argued of course that this absence of any increase in fatal bleeds from aspirin could be attributed to the deliberate selection of healthy individuals at low risk of bleeding for inclusion in clinical trials, yet this good clinical practice could also be widely adopted.

## 7. Some Further Questions??

Low-dose prophylactic aspirin raises important questions on public health policy:

- Is a distinction between the treatment of disease and the preservation of health valid? If it is, what are the responsibilities of the individual in relation to the second, and what are the responsibilities of healthcare practitioners – and in particular, public health practitioners? How is this debate to be resolved?
- Would the promotion of prophylactic aspirin, and the official ‘endorsement’ of the drug which promotion would imply, encourage an acceptance by lay people of a range of less efficacious and more dangerous medicines?
- Would the promotion of prophylactic drugs such as aspirin—but also statins and other prophylactics—be perceived as turning healthy people into patients? Do aspirin recommendations need to be revisited and revised [21].
- Bowel screening by testing for fecal occult blood and colonoscopy have been shown to reduce mortality, but the uptake of these procedures is low. Should aspirin be routinely used to enhance the effectiveness of cancer screening?

These are all important questions, and lay people should be involved with professionals in thinking through their implications. After all, in a globalised and increasingly affluent and well-educated world, we can be sure that individuals will increasingly access prophylactic medicines of all sorts, whatever their governments might wish, and these issues cannot be ignored. A further element to this is that media reports are already bringing the potential of aspirin into the wider domain.

## 8. Key Points

- Daily low-dose aspirin (75-100mg daily) reduces both vascular disease events and bowel and other cancers. A reduction in vascular disease events by aspirin is immediate but because aspirin works at a cellular level there is a 5-10 year delay before a reduction in cancer becomes evident.
- Aspirin increases the risk of intestinal bleeding, but the increase appears to be of less serious bleeds and there is no evidence of any increase in deaths from bleeding attributable to aspirin. So aspirin has a good safety profile.
- Recommendations for daily low-dose for all subjects aged over 50 years aspirin have been made and challenged. This remains an area of debate.
- There is an urgent need for wide discussions about how aspirin prophylaxis can best be handled. In particular, whether information on the risks and benefits of low-dose aspirin should be made widely available, within the context of healthy lifestyles, so that individuals can take informed decisions about the preservation of their health. The media is already playing a role.
- There is already a large amount of community use of aspirin. Efforts to boost usage in those recommended the medicine by a healthcare professional seem warranted. Those self-medicating with large doses could be given education and support to reduce the level to a more appropriate level.

## References

[1] Elwood P, Longley M (2010). My health: whose responsibility?: A Jury decides. J Epidemiol Commun H.

[2] Antithrombotic T (2009). Aspirin in the primary and secondary prevention of vascular disease: collaborative meta-analysis of individual participant data from randomised trials. Lancet.

[3] Elwood P, Morgan G, Fone D (2011). Aspirin use in a south-Wales county. Br J Cardiol.

[4] Morgan G (2012). Rapid health impact assessment of aspirin promotion for the secondary prophylaxis of vascular events in Wales. Qual Prim Care.

[5] Elwood P, Gallagher A.M, Duthie G.G (2008). Aspirin, salicylates and cancer. Lancet.

[6] Rothwell P.M, Wilson M, Elwin C.E (2010). Long-term effect of aspirin on colorectal cancer incidence and mortality: 20-year follow-up of five randomised trials. Lancet.

[7] Rothwell P.M, Fowkes F.G.R, Belch J.F.F (2011). Effect of daily aspirin on long-term risk of death due to cancer: analysis of individual patient data from randomised trials. Lancet.

[8] Burn J, Gerdes A.M, Macrae F (2011). Long-term effect of aspirin on cancer risk in carriers of hereditary colorectal cancer: an analysis from the CAPP2 RCT. Lancet.

[9] Phillips I, Langley R, Gilbert D (2013). Aspirin as a treatment for cancer. Clin Oncol (R Coll Radiol).

[10] Morgan G, Elwood P (2009). Could recommendations about aspirin prophylaxis enhance colorectal cancer screening programmes?. Eur J Public Health.

[11] Morgan G (2009). Aspirin for the prevention of vascular events. Public Health.

[12] Gorelick P.B, Weisman S.M (2005). Risk of haemorrhagic stroke with aspirin use: an update. Stroke.

[13] Hansson L, Zanchetti A, Carruthers S.G (1998). Effects of intensive blood-pressure lowering and low-dose aspirin in patients with hypertension: principal results of the Hypertension Optimal Treatment (HOT) randomised trial. HOT Study Group. Lancet.

[14] Elwood P, Morgan G, Brown G (2005). Aspirin for all over 50? For. British Medical Journal.

[15] Baigent C (2005). Aspirin for all over 50? Against. British Medical Journal.

[16] Baron J.A (2003). A randomized trial of aspirin to prevent colorectal adenomas. N Engl J Med.

[17] Chan A.T, Giconnucci E.L, Schernhammer E.S (2004). A prospective study of aspirin use and the risk for colorectal adenoma. Ann Intern Med.

[18] Brenner H, Tao S, Haug U (2010). Low-dose aspirin use and performance of immunochemical fecal occult blood tests. JAMA.

[19] Ajani U.A, Ford E.S, Greenland K.J (2006). Aspirin use among US adults: Behavioural Risk Factor Surveillance System. Amer J Prev Med.

[20] Sung J.J, Lau J.Y, Ching J.Y (2010). Continuation of low-dose aspirin therapy in peptic ulcer bleeding: a randomized trial. Ann Intern Med.

[21] US Preventive Services Task Force (2009). Aspirin for the prevention of cardiovascular disease: US preventive services task force recommendation statement. Ann Intern Med.

